# Antimicrobial Resistance: What Lies Beneath This Complex Phenomenon?

**DOI:** 10.3390/diagnostics14202319

**Published:** 2024-10-18

**Authors:** Giedrė Valdonė Sakalauskienė, Aurelija Radzevičienė

**Affiliations:** Institute of Physiology and Pharmacology, Medical Academy, Lithuanian University of Health Sciences, LT-44307 Kaunas, Lithuania; aurelija.radzeviciene@lsmu.lt

**Keywords:** antimicrobial resistance, multidrug-resistance pathogens, outer membrane vesicle, resistance mechanisms

## Abstract

Antimicrobial Resistance (AMR) has evolved from a mere concern into a significant global threat, with profound implications for public health, healthcare systems, and the global economy. Since the introduction of antibiotics between 1945 and 1963, their widespread and often indiscriminate use in human medicine, agriculture, and animal husbandry has led to the emergence and rapid spread of antibiotic-resistant genes. Bacteria have developed sophisticated mechanisms to evade the effects of antibiotics, including drug uptake limitation, drug degradation, target modification, efflux pumps, biofilm formation, and outer membrane vesicles production. As a result, AMR now poses a threat comparable to climate change and the COVID-19 pandemic, and projections suggest that death rates will be up to 10 million deaths annually by 2050, along with a staggering economic cost exceeding $100 trillion. Addressing AMR requires a multifaceted approach, including the development of new antibiotics, alternative therapies, and a significant shift in antibiotic usage and regulation. Enhancing global surveillance systems, increasing public awareness, and prioritizing investments in research, diagnostics, and vaccines are critical steps. By recognizing the gravity of the AMR threat and committing to collaborative action, its impact can be mitigated, and global health can be protected for future generations.

## 1. Introduction

The discovery and introduction of antibiotics from 1945 to 1963 marked a revolutionary period in medical history. However, as early as 1963, the first signs of antibiotic-resistant genes spreading across different bacterial strains through plasmids began to emerge [1,2]. Since 2013, antimicrobial resistance (AMR) has become a serious global threat comparable to climate change and the COVID-19 pandemic [1]. AMR occurs when bacteria changes render the drugs used to treat infections less effective or even ineffective. Recent data underscore the severity of this issue. In 2019, bacterial AMR was associated with an estimated 4.95 million deaths, including 1.27 million deaths directly attributable to it. Six leading pathogens—*E. coli*, *S. aureus*, *K. pneumoniae*, *S. pneumoniae*, *A. baumannii*, and *P. aeruginosa*—were responsible for 929,000 deaths attributable to AMR and 3.57 million deaths associated with it. Among these, methicillin-resistant *S. aureus* alone caused over 100,000 deaths in 2019, while other multidrug-resistant bacteria also contributed significantly to mortality rates [3]. The most alarming projection, established in 2014, estimated that AMR could cause 10 million annual deaths by 2050 [4]. This projection highlights the immense clinical and public health burden of AMR, though quantifying the excess morbidity and mortality associated with it remains challenging. Current global estimates, while concerning, indicate the urgent need for more detailed and reliable data to improve AMR control measures, preferably based on comprehensive, population-based surveillance from low-, middle-, and high-income countries [5].

In response to this growing threat, a high-level United Nations meeting is scheduled for September 2024 to discuss AMR and propose an enhanced framework beyond what was outlined in 2016. It is critical that world leaders and policymakers recognize the magnitude of this problem and ensure adequate global investment in developing innovative, affordable vaccines, antimicrobial agents, diagnostics, and reporting systems. The COVID-19 pandemic has highlighted the interconnectedness of our world—economically, culturally, and socially—where a crisis in one country can quickly impact others. Since December 2019, COVID-19 has caused an estimated 3.2 million deaths and cost the global economy approximately $17 trillion. On the current trajectory, by 2050, AMR could not only be responsible for up to 10 million deaths annually, but it is also projected to impose a staggering cost of over $100 trillion on the global economy. These figures far surpass the impact of COVID-19, highlighting the urgent and multifaceted threat that AMR poses [6]. Since the first antibiotic has been discovered, their widespread use in medicine and the agriculture industry for growth and prophylaxis has significantly contributed to the rise of multidrug-resistant infections. Today, numerous health concerns, including the treatment of immunocompromised patients, organ transplantation, and routine surgical procedures, are facing serious consequences due to AMR [1,2]. This review aims to provide a comprehensive understanding of the global drivers, factors, and origins of AMR, as well as its underlying mechanisms.

## 2. Phenomenon of AMR: Drivers and Risk Factors

**Figure 1:**
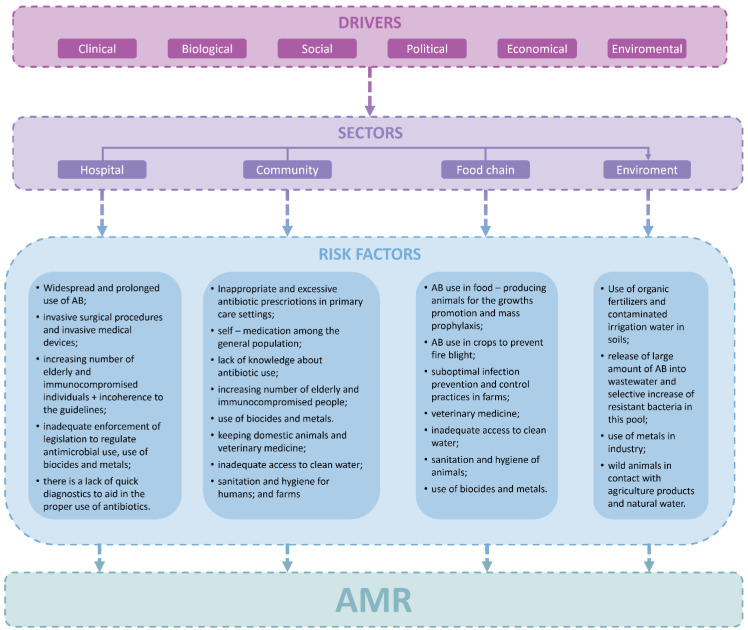
Framework of key drivers and risk factors in AMR [7,8,12,14,15,16,17]. AB—Antibiotics; AMR—Antimicrobial Resistance; +—plus.

AMR is predisposed by clinical, biological, social, political, economic, and environmental drivers. The presence of AMR bacteria in the environment is a consequence of the interactions among miscellaneous variables [7]. Thus, the main risk factors contributing to the emergence of AMR are classified into the following sectors: hospital, community, the food chain, including livestock and agriculture, and environment (Figure 1) [7,8]. Overuse and misuse of antibacterial agents are a core of this phenomenon. This harmful behaviour is often influenced by the general population’s poor or limited knowledge about appropriate antibiotic use and risk of AMR [7,9,10,11,12]. Other significant risk factor contributing to overuse and misuse of antibacterial drugs and consequently—AMR is inadequate adherence to the treatment and prophylaxis guidelines of infectious diseases in the primary care facilities and hospitals, e.g., incorrect duration of treatment, dosing and delay in the switch from intravenous to oral administration, erratic choice of spectrum with unnecessary broad-spectrum coverage, suboptimal infection prevention and control practices in health-care facilities (Figure 1) [7,8,12,13,14,15,16,17].

## 3. Origins of AMR

The origins of AMR are basically classified into three forms: natural (innate), acquired, and adaptive. Natural resistance may be intrinsic or induced (mediated). Intrinsic resistance is often genetically expressed within the bacterial species, does not depend on the contact with a specific antibiotic, and is not triggered by horizontal gene transfer. Induced resistance depends on the genes naturally occurring in the bacteria and activated to “insensitivity” levels only after exposure to an appropriate antibiotic. In other words, natural resistance mechanisms are genetically encoded in the bacterial genome and transmitted by “vertical gene transfer,” where genetic information, including any mutations, is transferred from “old” to “new” generations within a species [7,18,19,20,21,22].

**Figure 2:**
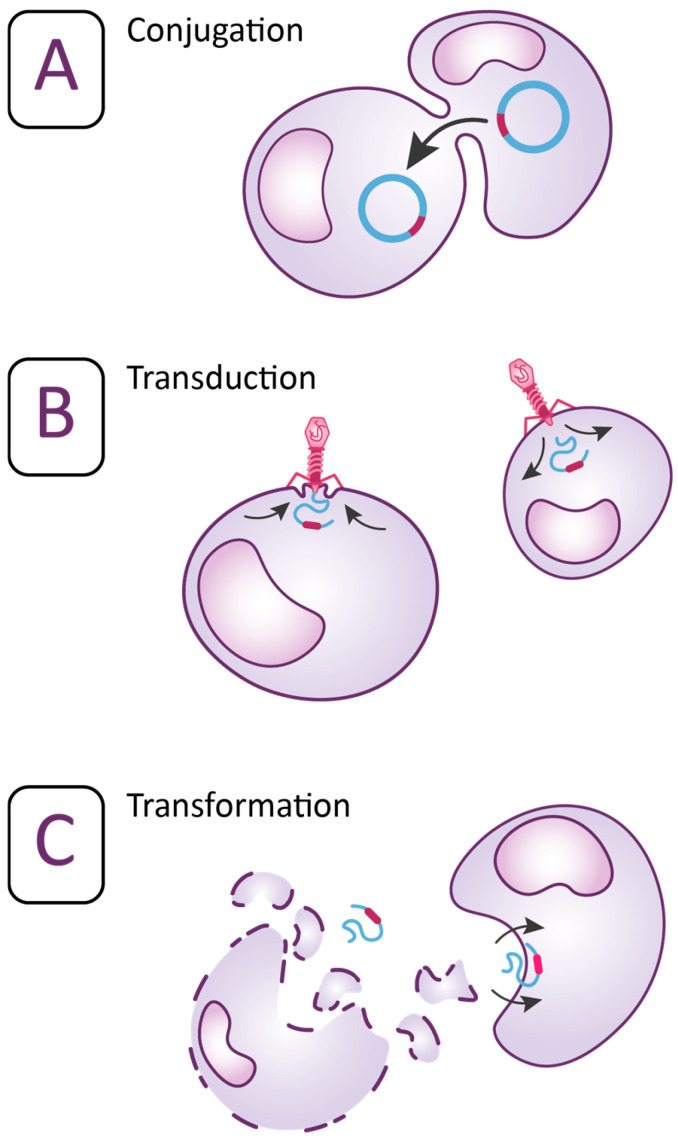
Mechanisms of “horizontal gene transfer” (**A**) represents the process when bacteria acquire resistance by transferring genetic material via conjugation. (**B**) represents the process when bacteria acquire resistance by transferring genetic material via transduction. (**C**) represents the process when bacteria acquire resistance by transferring genetic material via transformation [18,30,31].

Acquired resistance occurs as the result of an evolutionary process when the bacteria acquire genetic material by transformation, conjugation, and transduction. All these three pathways are called “horizontal gene transfer” (Figure 2) [20,21,23]. The process when the recipient bacterium takes up extracellular DNA released from donor bacterium is called transformation. Transfer of DNA from donor bacterium to recipient bacterium mediated by bacterial viruses is called transduction as the bacteriophage attaches to a receptor on the cell surface and introduces the DNA into recipient pathogen. The bacteriophages can also transmit plasmids from the donor bacteria to recipient bacteria. Plasmids are extrachromosomal DNA molecules common in many bacteria and can replicate independently from the chromosome [20,21,23,24,25]. However, the most common route to transfer resistance genes is conjugation. In conjugation, the donor bacteria transfer genetic material encoding AMR via the plasmids to the recipient pathogen by initiating the mating [20,21,23]. Aside the plasmids, other structures are involved in the development of AMR. These structures are mobile genetic elements (MGE): insertion sequences (IS), transposons (Tn), and gene cassettes/integrons (In). MGE can activate resistant genes in various species of bacteria, animal hosts, and the environment. They are responsible for the capture, accumulation, and dissemination of resistance genes. IS and Tn are discrete DNA segments that can move themselves and the associated resistance genes almost randomly to new locations in the same or different DNA molecules within a single cell. Integrons use site-specific recombination to move resistance genes between defined sites. MGE are often present in multiple copies in different locations of a genome, so they can also induce homologous recombination by exchanging sequences between identical or related segments. Intercellular mechanisms of genetic exchange are based on the classical pathways of “horizontal gene transfer” and different resistance genes can be transferred between bacteria belonging to the same or to different species [18,26,27]. Recent research has indicated that also “vertical gene transfer” contributes significantly to the formation of transconjugants and consequently spreading of AMR genes [28]. Bacteria may also acquire AMR via mutations in their own chromosomal DNA through five mechanisms: substitution, deletion, and addition of nucleotides or inversion and duplication of DNR segment in the genome [21,23,29]. Acquired resistance is usually permanent [27]. As a result of all these mechanisms, previously sensitive bacteria become nonresponsive to the antimicrobial treatment [20,21,23].

Adaptive resistance is induced by environmental changes and external stimuli that make an impact on bacterial growth factors, nutrition, or the environmental concentration of ions and pH or can provoke stress. It is usually obtained when bacteria are under exposure of subinhibitory concentrations of antibiotics. Contrary to the natural and acquired resistance, adaptive resistance is transient and generally reverts as soon as the causing signals disappear. Although the exact biological genesis of adaptive resistance has not been established yet, epigenetic inheritance, high mutation rates, gene amplification, efflux pumps, biofilm formation, bacterial swarming, the occurrence of persistence, population structure, and heterogeneity have been considered as the possible mechanisms for its development [18,19,23,32].

## 4. Mechanisms of AMR

**Figure 3:**
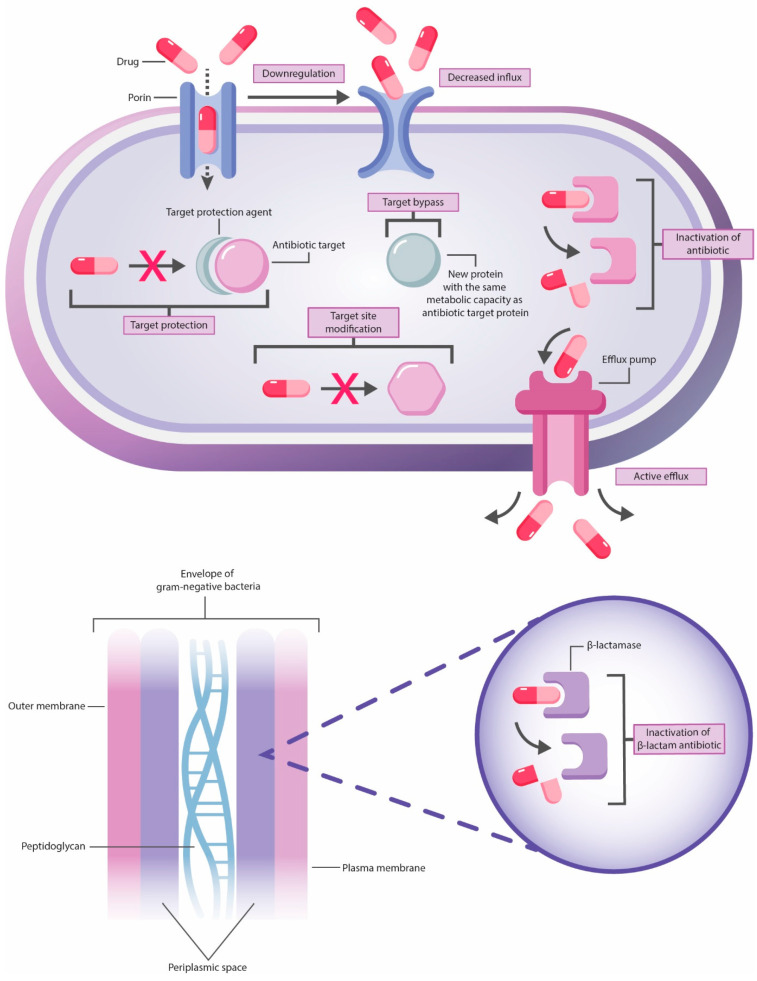
The main mechanisms of AMR.

The mechanisms of AMR are classified into four main categories: inactivation or alteration of antibacterial drug, modification of drug binding sites or targets, changes in bacterial cell permeability resulting in reduced intracellular accumulation of AB due to activation of efflux pumps, decreased regulation of porins expression or diminished pores’ width, and finally—biofilm formation (Figure 3) [18,20,33].

Intrinsic AMR is mainly attributable to the limited drug uptake, inactivation, and efflux from the cell while acquired—to drug target modification, inactivation, and efflux (Table 1) [21]. Gram-negative bacteria use all four resistance mechanisms. These pathogens have a hydrophobic outer membrane bilayer, containing lipopolysaccharides, phospholipids and outer membrane proteins, including pore–forming proteins—porins. Gram-positive bacteria due to the essential cell structure differences, especially lack of outer membrane, less commonly employ the mechanisms of limited drug uptake and drug expulsion related to the synthesis of certain efflux pumps. Thus, an outer membrane is an obstacle to several antibacterial drugs that are typically effective against gram-positive pathogens to reach the targets [21,23,34]. Multidrug-resistant *E. faecium*, *S. aureus*, *K. pneumoniae*, *A. baumanii*, *P. aeruginosa*, and *Enterobacter* spp. (MDR ESKAPE) pathogens have been sharing the same resistance mechanisms as other bacteria [33,35].

## 5. Biofilm Formation

Bacterial biofilms are communities of microorganisms that originate from single or multiple bacterial strains. These biofilms are commonly found on hospital instruments, body tissues, industrial surfaces, food processing units, and natural environments. Nearly all bacteria can form biofilms, which pose a significant challenge in treating bacterial infections and are a major cause of persistent infections. Biofilms exhibit increased resistance to conventional antibiotics and can cause device-related and tissue-associated infections, posing a severe threat to global health [43]. A biofilm consists of 10% biomass and 90% water. Approximately 50–90% of the organic components in a biofilm are polysaccharides known as exopolysaccharides, which are considered a major component of the extracellular polymeric substances (EPS) matrix. The polysaccharide chains form a dense, mesh-like structure, while positively charged ions create supportive cross-bridges between the polymers, helping biofilms maintain their thickness. In biofilms of Gram-negative bacteria, the polysaccharides can be neutral or polyanionic. It has also been established that biofilms can include uronic and mannuronic acids, as well as ketal-linked pyruvates, which provide anionic properties that contribute to the greater binding force of mature biofilms. Bacteria growing in biofilm are sessile and are responsible for most physiological processes in the biofilm environment. The sessile bacterial biofilm communities have different growth, gene expression, transcription, and translation rates. These functional characteristics are acquired by the sessile bacterial biofilm communities in the process of adaptation to microenvironments that have higher osmolarity, scarcer nutrients, and increased cell density. The resulting structure of a biofilm is extremely viscoelastic and has a rubbery behaviour [44]. Thus, biofilms can range from a few layers of cells to several centimetres in thickness, depending on environmental conditions. Formation of biofilm is a microorganisms strategy to protect themselves from other microbes, survive harsh conditions, and spread to new surfaces [18].

**Figure 4:**
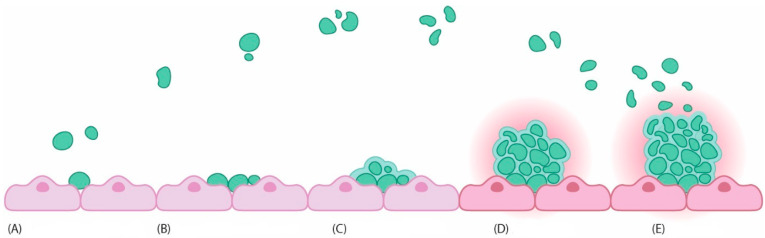
Stages of biofilm formation [43,44]. (**A**) Reversible attachment; (**B**) Irreversible attachment; (**C**) Growth and extracellular polymeric substances (EPS) production; (**D**) Maturation; (**E**) Dispersal.

The formation of biofilms is a multi-step process that involves initial attachment (reversible and irreversible) of single bacteria, bacterial aggregation and microcolony development, maturation and, finally, dispersion/detachment (Figure 4) [43,44]. The attachment on the specific surface of free-floating planktonic bacteria initiates the first stage. Attachment is driven by many factors such as bacterial appendage, e.g., pili, flagella, fimbriae, chemotaxis, surface properties of bacterial cell, sedimentation, material composition, temperature, and pressure. A crucial role in this process is played by the non-specific van der Waals and electrostatic forces, hydrophobic and steric interactions, and protein adhesion [43,44,45]. Attachment is initially reversible, mediated by weak van der Waals and electrostatic forces, as well as hydrophobic interactions, along with bacterial appendages. However, once bacteria adhere to a specific surface, adhesion becomes irreversible, leading to the formation of exopolysaccharides and the accumulation of multilayered cell clusters. It has been established that an intercellular signalling mechanism known as the quorum sensing system (QSS) also significantly facilitates the formation of bacterial biofilms from single cells.

Bacterial cells use the QSS to synthesize and release first messengers, such as chemical signals, to enable communication within the bacterial community. During this process, several physiological and structural changes occur, including the loss of motility in the adhered cells [43,45]. The subsequent stage following bacterial attachment involves cell aggregation, multiplication and division, leading to the formation of microcolonies. This process is driven by the extensive secretion of an EPS matrix enabled by specific chemical signals such as cyclic dimeric guanosine monophosphate (c-di-GMP). The secreted polymeric substances act as a binder that hold pathogens. EPS matrix plays a pivotal role in surface adhesion, in forming bacterial biofilms and internal biofilm structures, and in mutual cell recognition, signal transduction, nutrient acquisition, cell maintenance, and the exchange of genetic information. Consequently, bacterial colonies within a biofilm typically comprise diverse micro-communities. These micro-communities collaborate in various ways, facilitating substrate exchange, the movement of essential metabolic products, and the removal of metabolic waste. Biofilms create an optimal environment for syntrophic associations, where two or more metabolically distinct bacteria rely on each other to utilize specific substrates for their energy requirements [43,44,45]. In the next step, bacteria are progressing into the maturation stage. This phase is driven by attached cells releasing signalling molecules, following activating specific genes which are crucial for biofilm formation, and enhancing bacterial virulence by modifying gene expression. It begins with the secretion of EPS, stabilizing the biofilm structure and providing protection against antimicrobial agents. Over time, multiple layers of cell clusters accumulate and aggregate on the surface, eventually forming microcolonies embedded within the EPS matrix. Within these microcolonies, QSS and intercellular signalling play pivotal roles. Maturation proceeds in two distinct stages: Stage I involves cell-to-cell contact and the production of autoinducer molecules such as N-acylated homoserine lactone, while Stage II sees further expansion of microcolony size and thickness, typically reaching about 100 µm, marking the formation of well-established microcolonies. Bacterial interactions within the biofilm are characterized by cooperative associations, influenced by spatial proximity. During maturation, bacteria sense and respond to the spatial arrangement of neighbouring clusters, facilitating the formation of cohesive structures that efficiently bond with nearby cells. Overall, gene and protein expression within the biofilm is orchestrated at the collective level rather than individually. In essence, maturation encompasses EPS secretion, cell aggregation, chemical signalling, QSS mechanisms, and the development of both micro- and macro-colonies [44]. The final stage of bacterial biofilm development is the dispersal process, where released bacteria can spread to infect other parts of the organism and establish new biofilm structures.

The mechanisms of dispersal vary depending on the bacteria involved in the biofilm but typically include three main processes: detachment of bacterial cells from colony colonies, transfer of cells to other substrates, and attachment of cells to new substrates. Detachment from biofilms can occur actively or passively. Active detachment, known as seeding dispersion, involves bacteria releasing from the biofilm to adapt the environmental changes caused by matrix-degrading enzymes, antimicrobials, or nutrient scarcity. Passive detachment, termed shedding and erosion dispersion, occurs due to external forces. Lower levels of c-di-GMP can inhibit biofilm formation and promote its separation. The environmental factors such as temperature, pH, oxygen levels, and nutrient availability also influence the dispersal of bacterial biofilms [43]. Biofilm formation serves as a protective mechanism for bacteria against harsh environmental conditions, including antibacterial agents and disinfectants. The complex matrix of EPS in biofilms shields bacterial cells, preventing them from effective antibiotic penetration to bactericidal concentrations. Compared to planktonic bacteria, those within biofilms exhibit several-fold higher resistance to antibiotics, aided by the biofilm’s structure and physiological changes like slower growth rates. Key mechanisms contributing to biofilm resistance include the restriction of antibiotic diffusion by the polymeric matrix, antibiotic–matrix interactions that reduce antibiotic activity, enzyme-mediated resistance, altered metabolic activity, genetic changes, and efflux pump-mediated antibiotic expulsion. Biofilm-associated antibiotic resistance differs from innate resistance, involving unique molecular strategies such as antibiotic–matrix interactions, slow growth rates rendering antibiotics ineffective, genetic adaptations, and the formation of persisted cells tolerant to antibiotics. High mutation rates in biofilm-forming bacteria facilitate the development of resistance mechanisms, including the production of antibiotic-inactivating enzymes and persisted cells capable to survive in high antibiotic concentrations. Moreover, biofilms facilitate the horizontal transfer of resistance and virulence genes among densely packed bacterial populations, promoting genetic diversification and enhancing antibiotic resistance throughout microbial communities. Overall, the resistance of biofilms to conventional antibacterial agents stems from a combination of physical, physiological, and genetic factors, posing a significant challenge in combatting bacterial infections [45]. Given the well-established role of biofilms in AMR, it is crucial to explore novel strategies to combat these structures, including repurposing existing medications to target biofilms effectively. By studying bacterial populations that form biofilms, we can gain insights into AMR patterns, which is essential for developing more effective treatment plans.

The wound bed provides an ideal setting for microbes to adhere, colonize, and potentially cause infection, leading to a complex interaction between the host and the microbiome. Chronic wound infections often involve single or multiple microbial biofilms, making their management difficult due to these biofilms’ tolerance and resistance to antimicrobial treatments (such as systemic antibiotics or antifungals, as well as topical antiseptics) and the host’s immune defenses. Systemic antimicrobial therapy is typically not advised for chronic wound infections, and there is a lack of awareness regarding the potential benefits of using antimicrobial biomaterials as an alternative treatment option [46].

Moreover, it has been established that nanoparticles hold potential in the fields of biomaterials and drug delivery, providing innovative methods to address biofilm-associated infections. Therefore, there is a pressing need to develop bioinformatics tools that can analyse and predict AMR linked to biofilm formation [47].

## 6. OMV Formation

**Figure 5:**
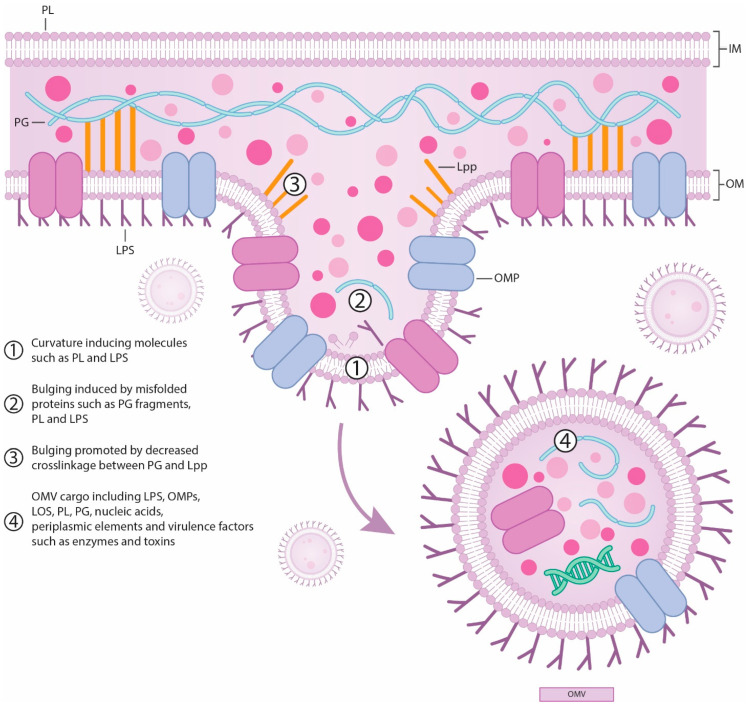
OMV production [50,51,52,53,54]. PL—phospholipids; LPS—lipopolysaccharides; PG—peptidoglycan layer; Lpp—lipoprotein that staples the outer membrane to peptidoglycan to maintain the structural integrity of the cell envelope; OMV—outer membrane vesicles; OMPs—outer membrane proteins; LOS—lipooligosaccharides.

Outer membrane vesicles (OMVs) are spherical bilayered nanoparticles derived from the outer layer of Gram-negative bacteria. While membrane vesicles are secreted by both Gram-positive and Gram-negative bacteria, the term “outer-membrane vesicles” specifically refers to those from Gram-negative bacteria that are naturally enclosed by an outer membrane. OMVs originate from the cell envelope of Gram-negative bacteria [48]. Gram-positive bacteria envelopes consist of the inner membrane (IM) and a peptidoglycan layer (PG), while Gram-negative cells are additionally encased in an outer membrane (OM). The OM is an asymmetric bilayer with the inner leaflet containing phospholipids (PL) and the outer layer primarily composed of lipopolysaccharides (LPS). Typically, LPS is composed of two phosphorylated glucosamines linked to multiple fatty acids, forming lipid A. This lipid A is attached to a short chain of species—specific sugars, known as core sugars, and a long chain of repeating sugar units is called the O-antigen. Proteins interacting with the OM are usually either beta-barrel proteins that span the membrane or lipidated proteins called OM lipoproteins. To maintain structural integrity, some outer membrane proteins (OMPs) and lipoproteins bind to the PG layer non-covalently. Additionally, the lipoprotein Lpp can create a covalent bond between these layers, effectively stapling the PG layers together (Figure 5) [49].

OMPs function as porins or serve structural roles. The space between the outer and cytoplasmic membranes, known as the periplasmic space, contains a thin PG layer and various periplasmic proteins. The PG layer acts as a protective skeleton for bacterial cells, guarding against osmotic and mechanical stresses. Periplasmic proteins are densely packed in this space, which is more viscous than the cytoplasm. The transmembrane Tol–Pal complex helps the OM span the periplasmic space and the cytoplasmic membrane [48]. The formation of OMVs is a complex process influenced by various factors, including environmental and host conditions. For instance, the enteric bacterial pathogen *C. jejuni* can detect host metabolites, such as sodium bile salts, which significantly impact OMVs production and content. When bile salts are present, they notably affect the genetic landscape involved in OMVs biogenesis, resulting in production of OMVs with a distinct protein profile compared to those produced without bile salts. This indicates a direct link between environmental factors and the genetic mechanisms controlling vesicle formation. It has also been observed that the presence of bile salts increases the mRNA transcription levels of serine protease genes related to OMVs biogenesis. Additionally, these conditions enhance the cytotoxicity and immunogenicity of OMVs towards intestinal epithelial cells [53]. Furthermore, use of antibiotics has shown the increase of OMVs production. Antibiotics such as ciprofloxacin, meropenem, fosfomycin, and polymyxin B induce OMVs production in *E. coli*, while carbapenems heighten OMVs secretion in multidrug-resistant *K. pneumoniae.* OMVs biogenesis begins with the outer membrane bulging and results in the release of vesicles into the surrounding environment. Several mechanisms have been proposed to explain OMVs production and regulation; however, a definitive mechanism has yet to be established [48]. The two widely accepted methods for OMVs formation are lytic (occurring during cell lysis) and non-lytic (occurring through outer membrane blebbing) [49]. The lytic method of OMVs formation involves the presence of PG residues containing autolysins within the OMVs. In this process, specific areas with higher concentrations of peptidoglycan during its synthesis cause the OM to bulge. This bulging initiates a series of signals that ultimately result in vesicle formation. Researches have shown that mutations in an autolysin involved in peptidoglycan replacement lead to increased OMVs synthesis, suggesting that the accumulation of peptidoglycan residues causes the OM to bulge, thereby facilitating vesicle release [53]. In contrast, the non-lytic method relies on four different pathways that involve the release of PG: reducing local OM–PG connections, increasing local OM curvature, raising periplasmic pressure, and flagellar release [49]. The covalent linkage of OM to PG is dependent on Lpp, which exists in equilibrium between PG-bound and unbound forms. A protein known as LdtF derived from *E. coli* and recently named DpaA can cleave Lpp–PG links. Disruption of Lpp’s PG binding function or deletion of *lpp* gene results in increased vesiculation. Even slight reductions in Lpp–PG linkage can induce vesiculation [49]. The limited number of lipoproteins binding to the PG layer can cause the OM to bulge, influencing the formation of vesicles [53,55].

The second pathway involves modifications to the OM bilayer. Various LPS modifications, such as mutations in LPS biosynthesis genes and deacylation of LPS fatty acids, are linked to OMV generation. Deacylation results in penta-acylated LPS, which increases membrane fluidity and promotes vesiculation, although some bacteria with only penta-acylated LPS still produce vesicles at reduced rates. Imbalances between the inner and outer OM layers, such as increased phospholipids (PL) in the outer leaflet of *H. influenzae* and *V. cholerae*, can also lead to vesiculation by decreasing OM stiffness, mimicking the effects of protein deletions or ethylenediaminetetraacetic acid (EDTA) treatment. Sulphur depletion in *N. meningitidis* may induce OMVs due to increased PL synthesis. Divalent cations like magnesium and calcium stabilize LPS and the OM, and EDTA, which chelates these cations, can lead to vesiculation and reduced OM rigidity. Similarly, negatively charged molecules like PQS (Pseudomonas Quinolone Signal) may decrease OM rigidity. *P. aeruginosa* OMVs are enriched in negatively charged B-band LPS, though strains lacking this LPS still produce OMVs with altered size and composition. Temperature changes also impact OMV production; increased temperature promotes vesiculation in *E. coli*, but not in *P. aeruginosa*, with opposite trends observed in *S. marcescens* and *B. henselae*. Bacteria may adjust membrane fluidity through fatty acid desaturation in response to temperature changes. Overall, vesiculation mechanisms are complex and may involve additional, less understood pathways [49].

Another pathway for OMV generation involves increased periplasmic pressure, which is triggered by the accumulation of misfolded proteins and other molecules within the periplasm. For instance, in *P. aeruginosa*, the depletion of a protein necessary for OMP synthesis leads to an accumulation of unfolded OMPs in the periplasm, which in turn enhances vesiculation. Interestingly, vesiculation can occur independently of periplasmic enrichment when OMPs are depleted. The deletion of periplasmic proteases, causing a buildup of misfolded proteins in the periplasm, also increases OMV production in this bacterium, independently of the PQS pathway. During membrane stress due to the accumulation of unfolded proteins in the periplasm, a small noncoding RNA that downregulates outer membrane protein A (OmpA) expression is produced, further increasing OMV generation. In *V. cholerae*, this process might result from a synergistic effect of increased periplasmic pressure from misfolded proteins and reduced OM—peptidoglycan connections due to decreased OmpA [49].

The fourth pathway is associated with flagellar release. Bacteria that have an LPS-sheathed flagellum can generate OMVs through flagellar rotation. These OMVs might be distinct from those formed via other pathways, as the proteome and lipid content of the flagellar sheath differ from those of the OM. In conclusion, the biogenesis of OMVs appears to involve multiple mechanisms that vary depending on species and growth conditions [49].

Significant progress has been made in understanding the genetic basis of OMV formation. In *S. typhi*, nine genes, collectively referred to as the “zzz genes”, have been identified as crucial for increased haemolysin E (HlyE) toxin secretion and OMVs’ biogenesis. The same genes are involved in protein and lipopolysaccharide synthesis responsible for various functions, such as envelope stability (*ompA*, *nlpI*, *tolR* genes), lipopolysaccharide synthesis (*rfaE*, *waaC* genes), peptidoglycan synthesis and remodelling (*mrcB gene*), stress sensing (*degS gene*), and global transcriptional regulation (*hns* gene) [53,56]. Once OMVs are released, they carry a diverse range of cargo, including LPS, OMPs, lipooligosaccharides (LOS), PL, PG, periplasmic elements, and virulence factors such as enzymes and toxins. OMVs have also been found to contain nucleic acids (DNA and RNA), although the packaging and delivery mechanisms of these nucleic acids are still under investigation. The molecular content of OMVs can vary depending on the bacterial species, growth conditions, and environmental factors. Proteins are among the most abundant components in OMVs and perform various functions. OMVs contain outer membrane proteins (OMPs), including porins such as OmpF, OmpA, and OmpC, which enhance bacterial adhesion to host tissues but can be recognized by specific receptors on host cells [53]. This adhesive capability is critical for bacterial infection and pathogenesis. OMVs allow bacteria to deliver virulence factors to distant sites while protecting these factors from degradation due to biochemical stress and avoiding direct cell-to-cell interactions. For example, *P. aeruginosa* OMVs carry multiple virulence factors that cause degradation and pore formation, exhibiting bacteriolytic effects on both Gram-negative and Gram-positive bacteria. The OMVs of *P. aeruginosa* and *B. fragilis* contain adhesion molecules such as aminopeptidase and hemagglutinin, respectively, which enhance bacterial adherence to host tissues.

OMVs play a role in immunomodulation. They are internalized by host epithelial cells through direct fusion or various endocytosis mechanisms. Following invasion, OMVs trigger inflammatory responses in epithelial cells, and increase levels of pro-inflammatory cytokines. OMVs contain components like LPS, flagellin, peptidoglycan, lipoproteins, DNA, and RNA, which act as pathogen-associated molecular patterns (PAMPs) that activate pattern recognition receptors (PRRs) in the host. The specific PRR signalling pathways activated by OMVs can differ between bacterial species. For instance, *E. coli* OMVs induce Toll-like receptor 4-dependent IL-8 production, while *N. gonorrhoeae* and *H. pylori* OMVs activate other PRRs, such as nucleotide-binding oligomerization domain-containing protein 1 (NOD1).

Inflammation induced by OMVs is not always beneficial for bacteria. It has been established that OMVs of *P. gingivalis* contain the cysteine proteinase gingipain, which degrades IL-8, while OMVs of *N. meningitidis* induce the production of anti-inflammatory cytokines such as IL-4, IL-10, and IL-13. However, *N. meningitidis* OMVs can both inhibit immune response and induce the production of pro-inflammatory cytokines, including IL-8, IL-1β, IL-6, and TNF. This demonstrates that OMVs play complex role in both inflammation and immunosuppression. Additionally, OMVs from commensal gut bacteria are thought to promote immune system maturation [48]. OM vesiculation functions as a response to envelope stress, aiding bacterial survival under challenging conditions [54]. Recent research has revealed that elevated levels of extra cytoplasmic function sigma factor or AlgU (also known as σ^H^), a homologue of σ^E^ or RpoE, an alternative sigma factor of *E. coli*, in *P. aeruginosa*, correlate with increased OMV production. Conversely, the loss of AlgU results in hypervesiculation, supporting the role of OMVs in alleviating envelope stress [54,57]. Additionally, OM vesiculation plays a role in managing oxidative stress. For instance, treatment with ciprofloxacin, which damages bacterial DNA and activates the SOS response, increases OMV production in *P. aeruginosa*. Mutants with impaired SOS responses also show reduced OMV production under antibiotic stress, suggesting that SOS response genes are integral to the vesiculation machinery. Moreover, hydrogen peroxide treatment significantly boosts OMV production, which relies on the bacterium’s ability to synthesize B-band LPS. B-band LPS is long and highly charged, and mutants unable to produce it exhibit impaired OMV production and increased sensitivity to oxidative stress [54]. OMVs from various bacteria, including *E. coli*, *P. syringae*, *V. cholerae*, and *M. catarrhalis*, have been shown to protect cells from host antimicrobial peptides and phage infections. They act as decoys, sequestering and binding these substances to protect the bacteria, effectively functioning as physical barriers that shield the bacteria from elimination [49,53,58,59].

OMVs are also crucial in neutralizing antibiotics and antimicrobial compounds. For example, OMVs from *M. catarrhalis* and *H. influenzae* interact with the complement system to reduce its activity, shielding other microbes. OMVs from *N. gonorrhoeae* bind and remove bactericidal factors from human serum, while *N. meningitidis* OMVs bind to neutrophil extracellular traps and bactericidal/permeability—increasing proteins, protecting the bacteria from these effects. Some bacteria produce OMVs that protect against membrane-dissolving agents and toxic levels of heme by binding these antimicrobial compounds, and effectively diluting their concentration. These protective effects are largely attributable to the OM composition and can be transferred to OMVs from other organisms [49]. OMVs are closely associated with AMR due to their role in transferring AMR genes. This AMR mechanism offers an alternative to traditional gene transfer methods, such as natural transformation, transduction, and conjugation, overcoming some of their limitations like host specificity, restricted genetic payload, and the type of genetic material transferred. OMVs can package and transfer genetic material, including antimicrobial resistance genes (ARGs), through horizontal gene transfer. This capability allows OMVs to carry drug-resistant genes across microbial communities, facilitating the acquisition of resistance genes from even distant relatives and contributing to the widespread dissemination of resistance among bacterial populations. This mechanism has been observed in several Gram-negative bacteria, including *A. baumannii*, *E. coli*, *P. gingivalis*, *N. gonorrhoeae*, and *P. aeruginosa*. Understanding the role of OMVs in AMR is crucial, as they accelerate the spread of resistance and complicate efforts to combat bacterial infection [53].

In addition to sequestering bactericidal compounds, OMVs can contain protective enzymes. For example, active β-lactamase has been found packaged in OMVs from *A. baumannii*, *M. catarrhalis*, *E. coli*, and *S. maltophilia* [49,53]. The encapsulation of β-lactamase in OMVs can protect the enzyme from inactivating antibodies and shield bacteria from β-lactam antibiotics [49,53]. OMVs from *S. maltophilia* not only protect this bacterium from β-lactam antibiotics but also confer resistance to other bacteria, such as *P. aeruginosa* and *B. cenocepacia*. Similarly, β-lactamase in OMVs from *M. catarrhalis* supports the survival of *H. influenzae* and *S. pneumoniae* [48,53]. OMVs from β-lactam-resistant *E. coli* degrade β-lactam antibiotics in a dose-dependent manner, rescuing β-lactam-susceptible *E. coli* and other bacterial species from antibiotic-induced growth inhibition [48]. Moreover, OMVs exhibit versatility in defending against various antibiotics, not just β-lactams. They significantly contribute to the degradation and neutralization of antibiotics such as colistin, melittin, and polymyxin. Some OMVs are enriched with catalase, an antioxidant enzyme that protects bacteria from oxidative damage, while others contain proteases that degrade proteins involved in active immunity and signalling [49]. OMVs also play a role in biofilm formation by packaging molecules that regulate both biofilm development and structure. For instance, OMVs from *P. aeruginosa* contain the quorum-sensing molecule PQS, which exerts an effect on biofilm structure and microbial diversity within the biofilm community. OMVs from *Aeromonas* strains can induce biofilm formation in a dose-dependent manner [53]. *P. gingivalis* OMVs play a significant role in bacterial aggregation and biofilm formation. This aggregation is thought to depend on gingipain proteases, which are enriched in OMVs and contribute independently to cell adhesion. Additionally, the iron uptake protein HmuY, targeted by *P. gingivalis* OMVs, has been implicated in biofilm formation. *P. gingivalis* OMVs also interact with extracellular DNA, potentially influencing the structure of the biofilm. These OMVs are involved in the formation of complex biofilms with *T. denticola* and *T. forsythia*. Moreover, *P. gingivalis* OMVs alone can induce the aggregation of various microbes within these complex biofilms, including pathogenic *S. aureus*. Other organisms within these biofilms, such as *T. forsythia*, also produce OMVs that aid in biofilm development. Thus, OMVs play a crucial structural role in the formation and maintenance of bacterial biofilms [49]. In addition to their significant role in antimicrobial resistance, OMVs have promising applications in biotechnology and medicine. These structures can be bioengineered and modified genetically or chemically to display specific antigens of interest. By expressing these homo- or heterologous antigens, OMVs can be used as vaccines to immunize individuals against various pathogens. Furthermore, OMVs have the capability to carry therapeutic cargo, making them an effective drug delivery system. Their inherent immunogenicity, stemming from proteins and glycans found on the outer membranes of Gram-negative bacteria, enhances their potential as tools for both vaccination and targeted drug delivery [52,60].

## 7. Conclusions

AMR is not merely a looming crisis but an active, global threat with far-reaching implications for public health, healthcare systems, and the global economy. As this article has explored, the roots of AMR are deeply embedded in the widespread and often indiscriminate use of antibiotics across human medicine, agriculture, and animal husbandry. The mechanisms that bacteria employ to resist antibiotics—ranging from drug degradation and target modification to efflux pumps, biofilm formation, and OMV production—are sophisticated and multifaceted, making the challenge of combating AMR complex and daunting. Addressing AMR necessitates a multifaceted approach. This includes not only the development of new antibiotics and alternative therapies but also a significant shift in how existing antibiotics are used and regulated. Equally important is the enhancement of global surveillance systems to accurately track and respond to the spread of resistance. Furthermore, public awareness and education about the responsible use of antibiotics are crucial in curbing the misuse and overuse that drive resistance. Policymakers must prioritize investment in research and development, not only for new drugs but also for rapid diagnostics and effective vaccines. By recognizing the gravity of the threat and committing to sustained, collaborative action, the impact of AMR can be mitigated, and global health can be protected for future generations.

## Figures and Tables

**Table 1:** Antibacterial agents, their mode of action and mechanisms of resistance [15,18,20,21,36,37,38,39,40,41,42].

Drug Class	Representatives (Drug Groups or Drugs)	Mechanism of Action	Drug Target	Mechanisms of AMR
Mechanism of action				
Inhibition of cell wall synthesis				
β lactams	Penicillins Cephalosporins Carbapenems Monobactams	Inhibition of cell wall synthesis	Peptidoglycan biosynthesis	Drug uptake limitation (decreased number of pores or changed selectivity of porins, no outer cell membrane) Enzymatic drug inactivation by hydrolysis (β lactamase production) RND ^1^ efflux pumps (reduction of drug absorption in the cell) Altered targets (PBP ^2^ alteration in gram-positive bacteria)
Glycopeptides	Vancomycin Teicoplanin		Peptidoglycan biosynthesis	Drug uptake limitation (thickened cell wall due to mutation, no outer cell wall or impermeable outer membrane in gram-negative bacteria) Altered targets (reprogramming of peptidoglycan biosynthesis via mutation of D-Ala-D-Ala ligase and peptidoglycan modification)
Disruption of bacterial cell inner membrane				
Lipopeptides	Daptomycin	Disruption of bacterial cell inner membrane	Inner membrane	Enzymatic drug inactivation by immobilization a hydrolytic cleavage of the ester bond between the threonine and kynurenine residue Altered targets (genetically determined alteration of the cell surface charge leading to the repulsion of the anionic daptomycin molecules, changes in the membrane composition via the alteration of phospholipid membrane metabolism, alteration of complex transcriptional regulatory networks governing the cell envelope stress response and membrane homeostasis, indirectly affected cell wall synthesis and consequently—thickness
Disruption of cell membranes’ structure, primarily the outer membrane, that finally causes cell lysis				
Cationic peptides	Colistin Polymyxin E	Disruption of cell membranes’ structure, primarily the outer membrane, that finally causes cell lysis ^1^	Cell membranes	Altered targets (overproduction of capsular polysaccharide, loss of LPSs ^3^ from bacterial outer membrane, modification of lipid A-pEtN ^4^ SMR ^5^ efflux pumps (reduction of drug absorption in the cell)
Aminoglycosides	Streptomycin Gentamicin Kanamycin Amikacin Tobramycin Spectinomycin	Inhibition of cytoplasm protein synthesis	Translation	Drug uptake limitation (cell wall polarity) Enzymatic drug inactivation (phosphorylation, acetylation, nucleotydilation) RND efflux pumps (reduction of drug absorption in the cell) Altered targets (ribosomal alteration due to mutation in 16S rRNA gene, methylation by the 16S ribosomal methylases)
Tetracyclines	Tetracycline Doxycycline Minocycline Tigecycline		Translation	Drug uptake limitation (decreased number of pores) Enzymatic drug inactivation (hydroxylation under FAD ^6^-dependent monooxygenases TetX ^7^ and TetX2 ^8^) Efflux pumps (reduction of drug absorption in the cell) Altered targets (ribosomal protection)
Macrolides	Erythromycin Azithromycin Clarithromycin		Translation	Enzymatic drug inactivation (hydrolysis, glycosylation, phosphorylation) ABC ^9^, MFS ^10^, RND efflux pumps (reduction of drug absorption in the cell) Altered targets (ribosomal mutation, 23S rRNA methylation, ribosomal protection by ABC ^11^ proteins)
Lincosamides	Clindamycin		Translation	Altered targets (ribosomal methylation due to modification of 23S rRNA by methyltransferases in gram-positive bacteria) ABC, RND efflux pumps (reduction of drug absorption in the cell) Enzymatic drug inactivation (phosphorylation, nucleotidylation)
Amphenicols	Chloramphenicol		Translation	Enzymatic drug inactivation (acetylation) MFS, RND efflux pumps (reduction of drug absorption in the cell) Altered targets (ribosomal methylation due to mutations within 23S rRNA of the 50S ribosomal subunit)
Streptogramins	Quinupristin and dalfopristin		Translation	Altered target (ribosomal alteration due to mutation of 23S rRNA in 50S ribosomal subunit) ABC efflux pumps (reduction of drug absorption in the cell) Enzymatic dug inactivation (acetylation, breakage of a carbo-oxygen bond by carbon-oxygen lyase)
Oxazolidinones	Linezolid		Translation	Altered target (ribosomal methylation by methyltransferases via modification of the 23S rRNA, ribosomal protection by ABC proteins) RND efflux pumps (reduction of drug absorption in the cell)
Fluoroquinolones	Ciprofloxacin Ofloxacin Levofloxacin	Inhibition of nucleic acid synthesis	DNA replication	Enzymatic drug inactivation (acetylation) MATE ^12^, MFS, RND efflux pumps (reduction of drug absorption in the cell) Altered targets (DNA gyrase modification in gram-negative bacteria, topoisomerase IV modification—in gram-positive, protection of DNA gyrase and topoisomerase IV)
Rifamycins	Rifampin		RNA synthesis	Altered targets (mutation of *rpoB* gene which encodes the β subunit of RNA polymerase) RND, SMR efflux pumps (reduction of drug absorption in the cell)
Pyrimidines	Trimethoprim	Inhibition of metabolic pathways (folic acid metabolism)	DHFR ^13^	RND efflux pumps (reduction of drug absorption in the cell) Altered targets (reduced binding and overproduction of DHFR due to modification or acquisition of novel DHFR genes)
Sulfonamides	Sulfamethoxazole		DHPS ^14^	RND efflux pumps (reduction of drug absorption in the cell) Altered targets (DHPS reduce d binding, overproduction of resistant DHPS due to mutations in the *DHPS* gene and *sul1/2* genes, which encode distinct DHPSs that are less susceptible to sulphonamide

^1^ RND—Resistance Nodulation and Cell Division Superfamily, ^2^ PBP—Penicillin binding proteins, ^3^ LPSs—lipopolysaccharides, ^4^ lipid A-pEtN—Lipid A phosphoethanolamine transferase, ^5^ SMR—Small Multidrug Resistance Superfamily, ^6^ FAD—Flavin adenine dinucleotide, ^7^ TetX—Monooxygenase conferring resistance to tetracycline antibiotics, ^8^ TetX2—Monooxygenase conferring resistance to tetracycline and tigecycline, ^9^ ABC—Adenosine triphosphate-binding cassette Superfamily of transmembrane transporters, ^10^ MFS—Major Facilitator Superfamily, ^11^ ABC proteins—Members of adenosine triphosphate-binding cassette F proteins, ^12^ MATE—Multidrug and Toxic Compound Extrusion Superfamily, ^13^ DHFR—Dihydrofolate reductase, ^14^ DHPS—Dihydropteroate synthase.

## Abbreviations

AB	antibiotic
ABC	Adenosine triphosphate-binding cassette Superfamily
AlgU	extra cytoplasmic function sigma factor
AMR	antimicrobial resistance
ARGs	antimicrobial resistance genes
c-di-GMP	cyclic dimeric guanosine monophosphate
COVID-19	coronavirus disease caused by the SARS-CoV-2 virus
DHFR	dihydrofolate reductase
DHPS	dihydropteroate synthase
DNA	deoxyribonucleic acid
EDTA	ethylenediaminetetraacetic acid
EPS	extracellular polymeric substances
FAD	flavin adenine dinucleotide
HlyE	haemolysin E
IL-1β	interleukin 1β
IL-4	interleukin 4
IL-6	interleukin 6
IL-8	interleukin 8
IL-10	interleukin 10
IL-13	interleukin 13
IM	inner membrane
In	gene cassettes/integrons
IS	insertion sequences IS
Lipid A-pEtN	lipid A phosphoethanolamine transferase
LOS	lipooligosaccharides
Lpp	lipoprotein
LPSs	lipopolysaccharides
MATE	Multidrug and Toxic Compound Extrusion Superfamily
MDR ĖSKAPE pathogens	multi-drug resistant ESKAPE pathogens indicated in the article
MFS	Major Facilitator Superfamily
MGE	mobile genetic elements
NOD1	nucleotide-binding oligomerization domain-containing protein 1
OM	outer membrane
OmpA	outer membrane protein A
OMPs	outer membrane proteins
OMVs	out membrane vesicles
PAMPs	pathogen-associated molecular patterns
PBP	penicillin-binding proteins
PG	peptidoglycan
PL	phospholipids
PQS	Pseudomonas Quinolone Signal
PRRs	pattern recognition receptors
RND	Resistance Nodulation and Cell Division Superfamily.
RNR	ribonucleic acid
RpoE	an alternative sigma factor of *E. coli*
QSS	quorum sensing system
SMR	Small Multidrug Resistance Superfamily
TetX	monooxygenase conferring resistance to tetracycline antibiotics
TetX2	monooxygenase conferring resistance to tetracycline and tigecycline
Tn	transposons
TNF	tumor necrosis factor
Comment: Names of bacteria and genes are presented in *italics*.	
